# Transporter annotations are holding up progress in metabolic modeling

**DOI:** 10.3389/fsysb.2024.1394084

**Published:** 2024-07-24

**Authors:** John Casey, Brian Bennion, Patrik D’haeseleer, Jeffrey Kimbrel, Gianna Marschmann, Ali Navid

**Affiliations:** ^1^ Biochemical and Biophysical Systems Group, Lawrence Livermore National Laboratory, Livermore, CA, United States; ^2^ Systems and Synthetic Biology Group, Lawrence Livermore National Laboratory, Livermore, CA, United States; ^3^ Earth and Environmental Sciences, Lawrence Berkeley National Laboratory, Berkeley, CA, United States

**Keywords:** metabolic modeling, transporter annotation, microbial community modeling, flux balance analysis, functional genomics

## Abstract

Mechanistic, constraint-based models of microbial isolates or communities are a staple in the metabolic analysis toolbox, but predictions about microbe-microbe and microbe-environment interactions are only as good as the accuracy of transporter annotations. A number of hurdles stand in the way of comprehensive functional assignments for membrane transporters. These include general or non-specific substrate assignments, ambiguity in the localization, directionality and reversibility of a transporter, and the many-to-many mapping of substrates, transporters and genes. In this perspective, we summarize progress in both experimental and computational approaches used to determine the function of transporters and consider paths forward that integrate both. Investment in accurate, high-throughput functional characterization is needed to train the next-generation of predictive tools toward genome-scale metabolic network reconstructions that better predict phenotypes and interactions. More reliable predictions in this domain will benefit fields ranging from personalized medicine to metabolic engineering to microbial ecology.

## 1 Introduction

Living systems interact with their surroundings. They acquire resources from their environment; co-operate, steal from, compete against, or kill their neighbors. Molecular compounds are the primary effectors of such interactions and thus the extent of these behaviors depend on the specialized transport proteins that move substances across membrane interfaces, into and out of cellular compartments. Microbes have designed transporters to access an incredible diversity of chemical species, enabling them to harbor pathways that generate cytotoxic byproducts (e.g., photorespiratory phosphoglycolate; Bauwe et al., 2012), to survive in harsh environments (e.g., acid mine drainage; Baker and Banfield, 2003), to harvest scarce resources (e.g., Lake Vostok, buried beneath 4 km of ice; Karl et al., 1999), to communicate with one another (e.g., quorum sensing in *Vibrio*; Hammer and Bassler, 2003), to attack one another (e.g., antibiotic production in soils; Chandra and Kumar, 2017), and to maintain a delicate balance of redox couples (Falkowski et al., 2008). For those interested in mechanistic modeling of such systems, knowing the full repertoire of microbial transport processes is crucial to predicting their dynamics in different habitats. This article describes the origins, state-of-the-art, challenges and future prospects of transporter functional annotation that we hope will serve as a “call to arms” for doubling efforts in both computational and experimental approaches.

Mechanistic, constraint-based modeling in systems biology has benefitted immensely from standardization of the model reconstruction process (Thiele and Palsson, 2010; Heirendt et al., 2019), testing and reporting the quality of models (MEMOTE; Lieven et al., 2020), consolidation of new algorithms and software into just a few dominant software platforms (overwhelmingly COBRA; Ebrahim et al., 2013; Heirendt et al., 2019), and sharing in just a few dominant formats (overwhelmingly SBML; Keating et al., 2020). That coordination has paved the way for an ever-growing and active community of software developers, engineers, systems biologists and computational biologists working to relax many of the rigid assumptions of the first generation of flux balanced models (Varma and Palsson, 1994). While the software and protocols are fairly thorough, there are several aspects of model reconstruction that are a bit flimsy, including what to do about polymers, quinones, and, as we discuss in detail here, transporters. Some authors may take the effort to report what those decisions were and why they were made, but there is certainly space for our community to weigh in on these persistent concerns.

The accuracy of genome-scale metabolic model (GEM) predictions are strongly correlated to the quality and completeness of the metabolic network reconstructions (Bernstein et al., 2023). The availability of transport mechanisms for import of nutrients greatly influences choice of gap-filled reactions in both automatically generated and curated models. This issue is further complicated by the “moonlighting” nature of some proteins (Jeffrey, 2018) where under different conditions they assume different functional roles. Many proteins also exhibit weak promiscuous activities for a variety of metabolites which leads to an “underground metabolism” that plays a major role in the fitness of organisms (Noterbaart et al., 2018). Not accurately accounting for presence of some imported metabolites will lead to exclusion of these reactions from the final network reconstruction and could lead to errors in assessing the robustness of a system to various types of perturbation. In previous work we have shown that functional annotation tools generate metabolic annotations that are incomplete and inconsistent with each other, and that the same is true for transporter annotations, with typically less than half the transporter annotation tools having substrate predictions that are sufficiently detailed to be incorporated in a metabolic model (Griesemer et al., 2018).

## 2 Discussion

### 2.1 Transporter annotations: what could go wrong?

**FIGURE 1:**
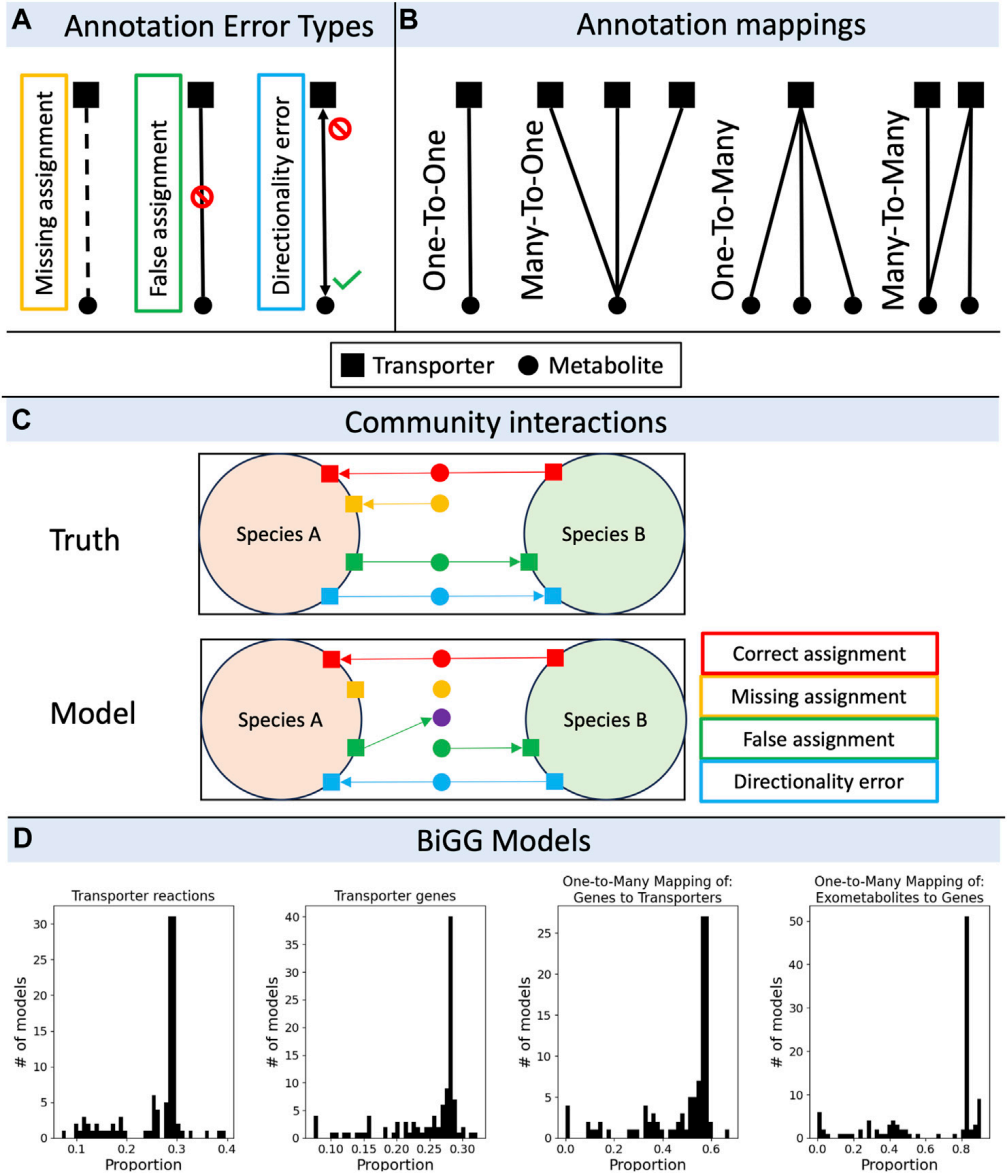
The pitfalls of transporter annotations in community metabolic modeling. **(A)** Types of errors encountered when assigning a single putative transporter to a single substrate. An annotation may miss an assignment where there should be one, may create an assignment where there should not, or may get the direction(s) of transport wrong (either due to an incorrect orientation of an irreversible process, or due to a reversibility error). **(B)** Mappings from transporter genes to substrates are non-unique. A single gene may map to a single substrate or multiple substrates, a single gene may be a part of a complex with multiple genes which map to a single substrate or multiple substrates. **(C)** Microbial interactions are variously affected by transporter annotation errors. For example, a species might not grow with missing assignment errors, the community might accumulate or deplete extracellular metabolites by false assignment errors, or a mutualism might be broken by directionality errors. **(D)** Analysis of transport mappings in BiGG models (n = 108 models). Histograms showing the proportion of transporter reactions to total reactions (left), the proportion of transporter genes to total genes (second from left), the proportion of one-to-many gene-to-transporter mappings to total transporter genes (second from right), and the proportion of one-to-many exometabolite-to-transporter gene mappings to total exometabolites (right). The large peaks correspond, mostly, to models of *Escherichia coli*, which are overrepresented in the BiGG database.

Pitfalls in matching transporters to their substrates come in a variety of flavors. We define three elemental error types—missing assignments, false assignments, and directionality errors (Figure 1A). There may be a fourth, somewhat more esoteric error type not included in the figure that applies to the case of a transporter that modifies a substrate during import (e.g., the phosphotransferase complex). These are likely rare and we have not encountered one, but an error in the annotation of the substrate modification or choice of cofactor (e.g., symporters) could conceivably occur. The frequency of different error types is likely variable for different species and for different annotation tools, but for some approximate context we quantified these errors in the model organism *E. coli* K12 MG1655, comparing an extensively curated GEM (iML1515; Monk et al., 2017) against an automatically generated GEM for the same genome using CarveMe (v1.5.2; Machado et al., 2018). Although transporter annotations in iML1515 may be updated in the future, we consider it a high-quality benchmark for evaluating error rates in automatically generated GEMs. In the CarveMe draft model, missing assignments accounted for 8.9%, false assignments accounted for 16.2%, and directionality errors accounted for 4.5% of the total transport reactions. Thus, nearly a third of annotated transporter functions were in error; because this strain is massively overrepresented in the BiGG database (King et al., 2016) that CarveMe references, we should treat these error rates as an underestimate of the error rate expected for non-model organisms using the same method. Griesemer and others showed that genome coverage by metabolic annotation tools, and discrepancies in annotation across different tools are significantly worse for organisms that are more phylogenetically distant from well-studied model organisms such as *E. coli* and *B. subtilis*, and we expect the same to be true for transporter annotations (Griesemer et al., 2018).

Each error type applies in GEMs to four types of gene-protein-reaction (GPR) mappings—one-to-one, one-to-many, many-to-one, and many-to-many (Figure 1B). Non-unique mappings between transporter genes, transporter proteins, and substrates arise from the possibility that individual transporters have more (one-to-one) or less (one-to-many) specificity in binding or selective permeability, and that individual substrates may bind or pass through one (one-to-one) or more (many-to-one) transporters. An analysis of all manually curated models in the BiGG database (King et al., 2016) revealed a wide range of unique mapping frequencies, with 36% ± 29% (range 0%–91%) of exometabolites mapping uniquely to a single transporter gene (*n* = 108 models; Figure 1D). As an added layer of complexity, gene products may be associated with more than one transporter complex (e.g., the GLUT1 subunit is present in multiple sugar transporters), which themselves may have broad substrate specificity (many-to-many) or serve as a common structural protein for various transporters. As we explore sources for the different error types and how those errors propagate through non-unique mappings in more detail (Figure 1C), it is worth reviewing the current state-of-the-art in automated functional transporter annotation tools and the databases they reference to address these pitfalls.

### 2.2 Transporter annotation tools and databases

Besides the major sequence repositories, there are currently two primary online database resources dedicated to transporters, and several more niche databases which focus on specific taxonomic groups or transporter types (Table 1). With two decades of development and curation, the Transporter Classification Database (TCDB; Saier, 2006; Saier et al., 2009; Saier et al., 2014; Saier et al., 2016; Saier et al., 2021) remains a central clearinghouse for transporter structures, bioinformatics tools, and is the official home of the Transporter Classification (TC) system ontology, a scheme based on mechanism, energy source, taxonomy and substrate. Since 2001, the International Union of Biochemistry and Molecular Biology (IUBMB) has designated the TC system as the formally recognized ontology for membrane transporters across all domains of life (Busch and Saier, 2003). Each entry in TCDB is manually curated and often accompanied by a detailed summary of the literature, and is maintained by a well-known authority on transporters. Surprisingly, Kroll and others reported that more than half of TCDB entries scored poorly (2 or below, on a scale from 1 to 5) on the UniProt annotation scale, and instead opted to rely on GO and UniProt entries (only those with a score of 5; Kroll et al., 2023). TransportDB (now in version 2.0; Elbourne et al., 2017) is another popular resource for systems biologists which builds on the TCDB and NCBI datasets, with entries currently available for 2,761 organisms (predominantly bacteria, though there are some eukaryotes and archaea) through a graphical and convenient web-portal. Entries in TransportDB are computationally derived with their accompanying annotation tool called TransAAP.

**TABLE 1:** Databases dedicated to transporters. NA, URL not maintained.

Database	Description	URL	Reference
ABCdb	Prokaryotic ATP binding cassettes. Curated and computational partitions	www-abcdb.biotoul.fr/	Fichant et al. (2006)
ARAMEMNON	Plant membrane proteins. Computational	aramemnon.botanik.uni-koeln.de/	Schwacke et al. (2003), Schwacke and Flügge (2018)
TCDB	All transporters. Curated	www.tcdb.org/	Saier (2006), Saier et al. (2009), Saier et al. (2014), Saier et al. (2016), Saier et al. (2021)
YTPdb	Yeast membrane proteins. Curated	NA	Brohée et al. (2010)
TransportDB 2.0	All transporters. Computational	http://www.membranetransport.org	Elbourne et al. (2017)

A chronology of transporter annotation tools, their various approaches, and a summary of their performance is available elsewhere (Alballa et al., 2020; Cunha et al., 2023), and we simply provide a convenient lookup table with short descriptions and URLs for reference (Table 2). Recently, the TranSyT tool (Cunha et al., 2023) has emerged as a front-runner alongside TransAAP. In the spirit of integration and ease of use, TranSyT can be implemented as a standalone app to generate a SBML file of transport reactions, or within popular automated GEM reconstruction pipelines like Merlin (Capela et al., 2022) and the ModelSEED reconstruction tools in KBase (Faria et al., 2023). TranSyT also scores annotations, a feature which may be leveraged for merging multiple annotation sources (Henry et al., 2010; Greisemer et al., 2018) or for generating ensemble GEM reconstructions.

**TABLE 2:** Annotation tools dedicated to transporters. Note that some portals appear to no longer be maintained (NA), while others have changed URLs since publication.

Name	Notes	URL	Reference
TransAAP	Integrated with TransportDB	www.membranetransport.org/	Elbourne et al. (2023)
TIP	Integrated with PathwayTools; parses existing text-based annotations	bioinformatics.ai.sri.com/ptools/	Lee et al. (2008), Karp et al. (2020)
TrSSP	Standalone, SVM annotation	www.zhaolab.org/TrSSP/	Mishra et al. (2014)
TRIAGE	Formerly the annotation tool for Merlin	NA	Dias et al. (2017)
TransATH	Standalone, automated pipeline based on Saier’s protocol	NA	Aplop and Butler (2017)
TranCEP	Standalone, combined homology and SVM annotation	github.com/bioinformatics-group/TranCEP	Alballa et al. (2020)
TranSyt	Successor to TRIAGE, standalone and integrated with Merlin, KBASE	transyt.bio.di.uminho.pt/	Cunha et al. (2023)
TransportTP	Standalone, combined homology and SVM annotation	NA	Li et al. (2009)
PortPred	Standalone. Combined DL-based protein embeddings and ML classification	github.com/MarcoAnteghini/PortPred	Anteghini et al. (2023)
SPOT	Standalone. DL using Transformer Networks for classification of transporter-substrate vector pairs	github.com/AlexanderKroll/SPOT	Kroll et al. (2023)

### 2.3 Modeling microbial community interactions

Genome scale models have been used in simulating microbial interactions for nearly two decades (reviewed by Heinken et al., 2021), and numerous algorithms have tackled the problem from different angles (reviewed by Biggs et al., 2015; Bauer and Thiele, 2018; Deiner and Gibbons, 2023; Scott et al., 2023). The architecture of community models, whether they ought to be compartmentalized or pooled into a “super-organism,” and whether one should attempt to sample the combinatorial interactions with flux balance analysis or to isolate the elementary modes of exchanges was pondered early on (Taffs et al., 2009; Perez-Garcia et al., 2016). Common to most of the more recent attempts is a compartmentalized approach with either stationary or dynamic flux balance analysis, wherein each strain-specific model interacts through an extracellular “compartment” through the exchange of metabolites. Intuitively (and formally; Klitgord and Segre, 2010), the compartmentalization of pathways, or parts of pathways, or of entire metabolic networks strongly influences predicted flux distributions and interactions. For example, a non-compartmentalized model might regenerate ATP from ADP in the absence of a proton motive force. Thus, an accurate accounting of which substrates, which products, and which reactions are where is vital to constraining fluxes and identifying modes of species-species interactions within a community.

Automated reconstruction of draft GEMs has improved considerably over the past decade (Machado et al., 2018; Wang et al., 2018; Heirendt et al., 2019; Faria et al., 2023), making great strides in closing the gap with curated models from genome information alone, but a recent analysis of automated and non-gapfilled draft GEMs showed dismal performance in predicting substrate utilization (Gralka et al., 2023). While there is still no substitute for manual curation by a skilled hand, draft GEM quality could be markedly improved through more comprehensive transporter annotations (Zuniga et al., 2021). Expansion from monoculture simulations to more complex communities likely amplifies these errors, resulting in poor agreement between predicted and actual growth rates in a gut community using three of the latest community FBA algorithms (Pearson correlations of 0.07, at best; Joseph et al., 2024). Special attention to microbial interactions (Sung et al., 2017) was given in the AGORA bacteria reconstructions (Magnúsdóttir et al., 2017; Heinken et al., 2023) and for the human host (the number of extracellular transport reactions ballooned from 537 in Recon1 to 1,537 in Recon2; Sahoo et al., 2014), but clearly there is room for more accurate and comprehensive representation of transport processes to improve growth and interaction predictions.

### 2.4 Challenges for transporter annotation databases and tools

Guiding principles from the larger systems biology community of shared access, integration and formatting, consistent with the FAIR principles (Barker et al., 2022), should be adopted when building relational databases and the tools that draw from them. This includes providing persistent link identifiers for genes, proteins, and substrates to common resources (e.g., NCBI, PubChem, BRENDA, RHEA) wherever possible, providing documented API’s for user access, adhering to community standard formats like SBML and JSON, in the case of tools, working with other developers to integrate with community standard reconstruction pipelines like COBRA and KBase. As we look to the next-generation of transporter annotation tools, especially those that build from emerging methods in machine learning and artificial intelligence, databases that prioritize these principles will be more readily accessed and leveraged.

Database and tool developers should also seek to provide, wherever possible, a minimal set of functional attributes of transporter gene annotations required for GEM reconstruction. We have identified five such attributes: membrane localization, membrane orientation (inward vs. outward facing), binding reversibility, substrate specificity, and reaction stoichiometry. We will discuss the current approaches and challenges in assigning these attributes.

#### 2.4.1 Membrane localization

With the exception of a few exceptionally well-studied model organisms, protein localization across an entire proteome, or even a substantial portion, is typically unknown *a priori*. A number of predictive tools are based on homology to manually curated databases of proteins of known localization (e.g., PSORT; Yu et al., 2010) or based on identification of transmembrane domains and their orientation (e.g., TMPred; Cuthbertson et al., 2005). Today, 77 protein subcellular localization prediction tools are now listed in bio.tools (reviewed in Li et al., 2023), with the newest generation (e.g., TmAlphaFold; Dobson et al., 2023) taking advantage of recent advances in structural prediction. Several are tailored to specific model organisms, while others draw from a broader taxonomic resolution. In the absence of sanity-checks for each compartmentalized reaction during the reconstruction process for a particular species, and given the importance of assigning transporters to the correct membrane, it may be wise to consider a consensus localization (e.g., COMPARTMENTS; Binder et al., 2014) from a collection of the most relevant sorting tools and other sources.

#### 2.4.2 Transporter orientation and reversibility

Secondary-active transporters like ion symporters and antiporters are typically reversible, but are often practically irreversible under physiological conditions. However, a famous counter-example is the oxygen-dependent transport of glutamate into and out of nerve cells (Szatkowski et al., 1990). Even in this non-canonical case, forward and reverse kinetics may be radically different for inward- and outward-facing protein orientations (Zhang et al., 2007). Primary-active transporters are, to our knowledge, strictly irreversible. Because of its functional classification scheme, annotation to the TC ontology should cover all but the most egregious cases of reversibility.

#### 2.4.3 Substrate specificity

**FIGURE 2:**
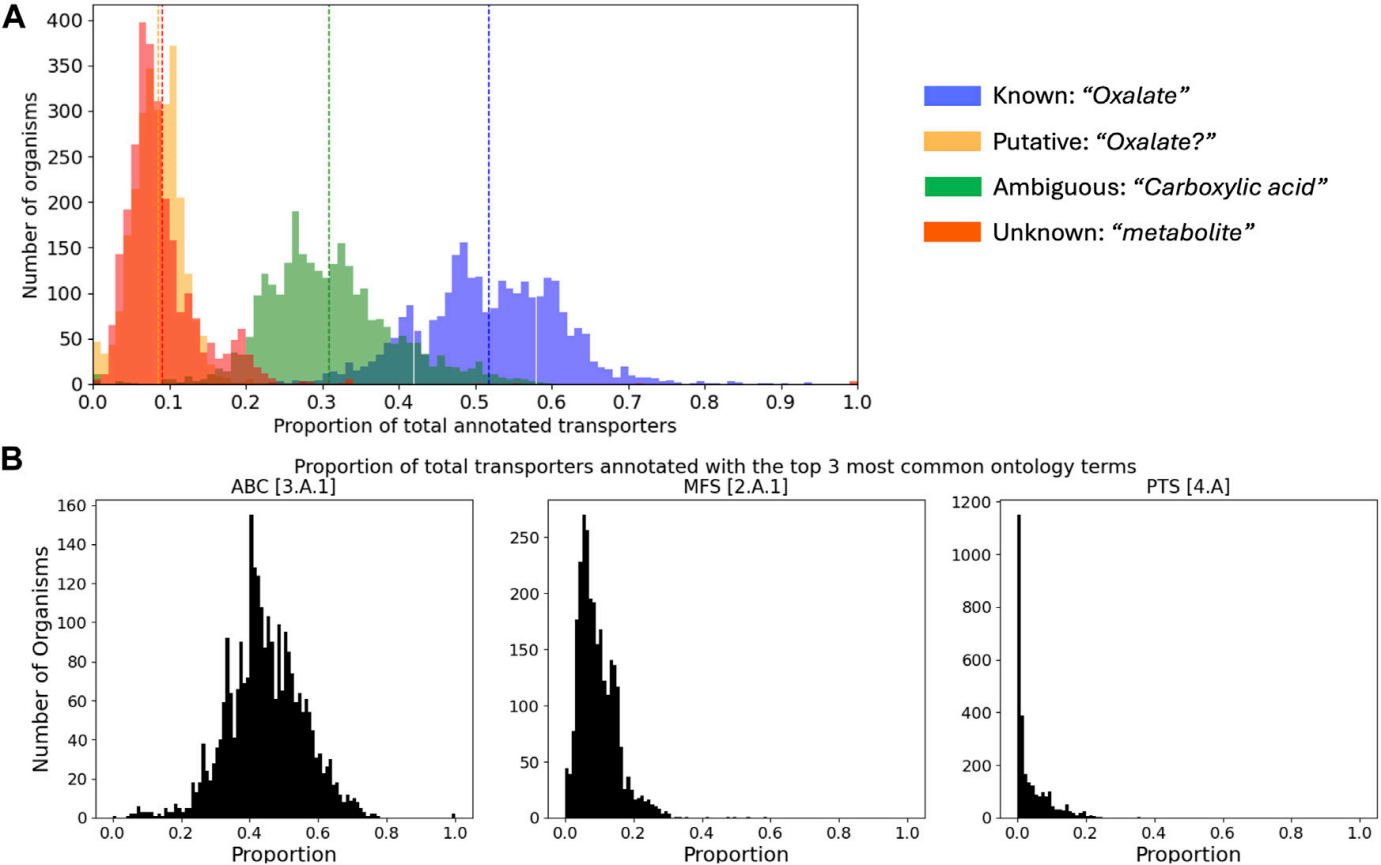
Summary of transporter annotations retrieved from TransportDB 2.0. **(A)** Distributions of the proportion of transporters annotated to different levels of specificity across all organisms. Vertical dashed lines correspond to the mean of each distribution, and an example of each category is provided. **(B)** Distributions of the proportion of transporters of the top 3 most abundant [super-] families across all organisms. ABC–ATP binding cassette; MFS–major facilitator superfamily; PTS–phosphotransfer-driven group translocators.

Because assigning substrates to transporters is the crux of the matter, we conducted an analysis of TransportDB 2.0 (Elbourne et al., 2017), the most extensive database of transporter annotations currently available. The dataset comprised 2,661 unique substrate names associated with 940,581 substrate-transporter pairs, distributed among 2,745 organisms. Substrates link identifiers were unavailable, and a single substrate often appeared with multiple names (e.g., “sodium ion” vs. “Na+”), making an estimate of the true number of unique substrate-transporter pairs difficult. For a subset of the unique substrate names (for practical reasons, those which appeared in more than 8 organisms), we manually assigned substrates into four categories: known (e.g., “Oxalate”), putative (containing a “?”; e.g., “Oxalate?”), ambiguous (“a carboxylic acid”), and unknown (e.g., “metabolite”). From this categorization across all organisms, we found that 52% ± 9% were known, 9% ± 4% were putative, 31% ± 8% were ambiguous, and 9% ± 6% were unknown (Figure 2A). Although the full 5-level TC system ontology terms are returned with TransAAP, the datasets available through TransportDB 2.0 contain only the first three levels (194 unique terms). From this coarse resolution, we found that only 5 ontology terms represented a majority (66% ± 9%) of all transporter annotations across all organisms, with a single term (3.A.1; ATP binding cassettes) representing nearly half (45% ± 11%; Figure 2B).

A single transporter may have similar affinity for multiple compounds, or even entire classes of compounds. This means that in some cases, a transporter might be annotated to an ambiguous level of substrate specificity (e.g., “a dicarboxylate”) not because of a lack of knowledge of the appropriate dicarboxylate molecule it transports (annotation is a missing one-to-one mapping), but rather because it has broad specificity for multiple dicarboxylate molecules (annotation is truly a one-to-many mapping); perhaps even with comparable kinetic properties. Modest changes of just one or two residues in transporter binding domains can affect substrate specificity and even stoichiometry, as is the case for the cation/proton antiporters (Masrati et al., 2018), so degeneracy in substrate specificity might be unfortunately necessary.

### 2.5 The trouble with diffusion

Although the selective permeability of membrane lipids with different lipid compositions have been described in great detail (Hannesschlaeger et al., 2019), diffusion reactions beyond the gasses and a few waste products are rarely included in GEM reconstructions. This may partly be due to the arbitrary nature of delineating the broad spectrum of diffusion rates, from fast (order 10^–2^ m^2^ s^−1^; e.g., oxygen) to slow (10^–10^ m^2^ s^−1^; e.g., high molecular weight polar compounds) diffusing molecules. In general, phosphorylated metabolites might be considered slow, eliminating a sizable portion of the total intracellular metabolites, but the line becomes blurry when considering small nonpolar metabolites like fatty acids, alkanes or alcohols. To make matters worse, the decision to include a diffusive reaction for a metabolite which is also actively transported would result in an underestimate of energy costs in standard FBA. In addition to specificity in transmembrane permeability, diffusive transport across other intracellular compartments, like the shell proteins of cyanobacterial carboxysomes which show preference for negatively charged ions (Mahinthichaichan et al., 2018), should be represented. Knowledge of the localization of pathways, or parts of pathways within, can aid in filtering the list of candidate diffusive reactions into and out of subcellular compartments, but this area is ripe for progress.

### 2.6 Prospects for computational approaches to transporter functional annotation

The state-of-the-art in transporter annotation brings together sequence alignment, systems biology ontologies, and structure analysis to make predictions about whether a gene product is a transporter, where it might be located, its orientation, and what substrates it might bind. Nevertheless, we find that many transporters lack sufficient coverage in one or more of the required attributes. A leap forward will address gene-protein-reaction specificity first.

**FIGURE 3:**
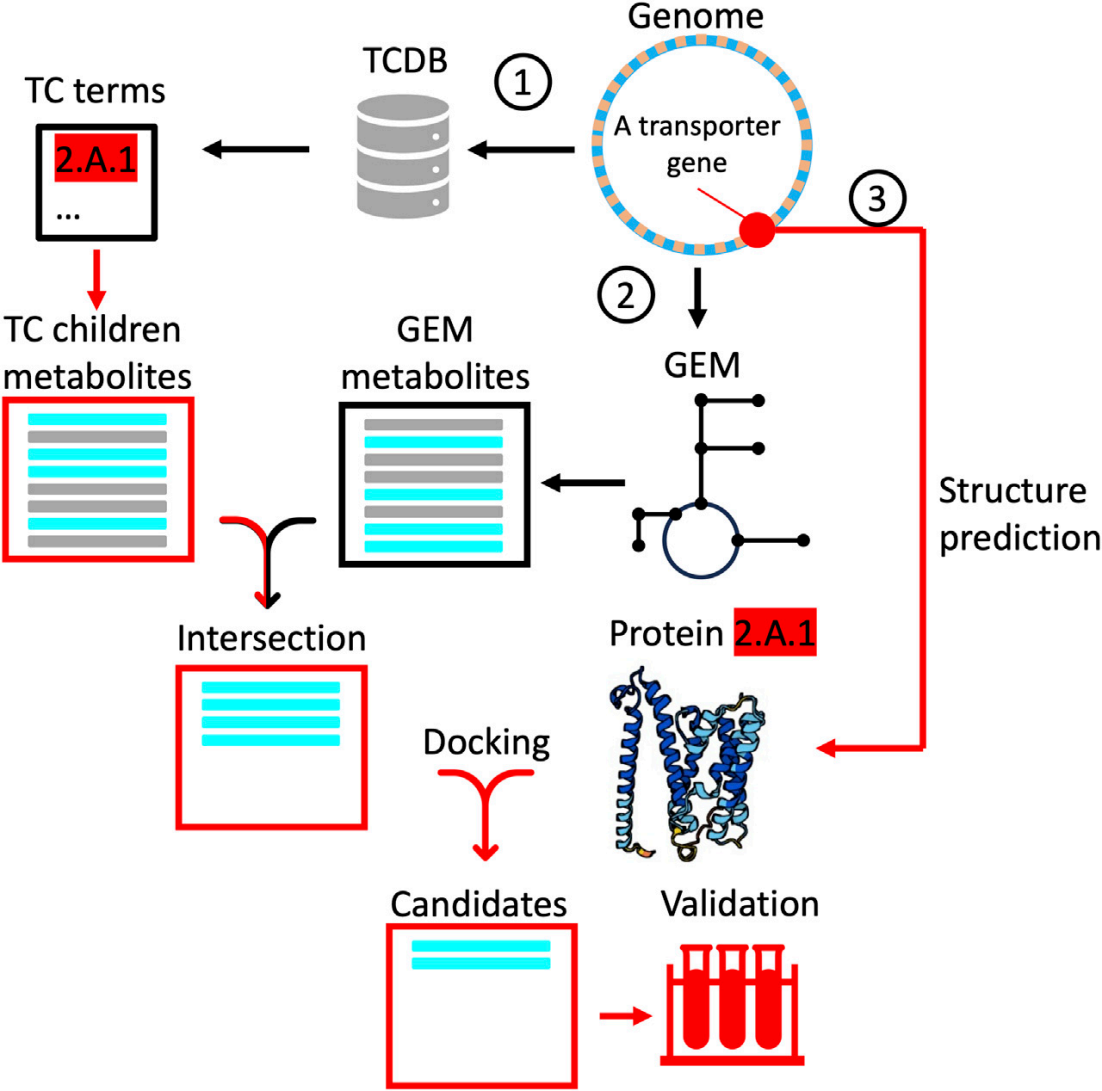
A proposed computational workflow to progressively narrow the search space for experimental validation of transporter functional annotations. Red lines correspond to paths followed for a single transporter and are repeated for all un-annotated transporters, while black lines correspond to paths taken (once) for the whole genome. The pipeline begins (1) with alignment of transporter genes to the TCDB, retrieving a list (horizonal bars) of all children metabolites associated with the lowest common ancestor ontology term. In another path (2), a draft GEM is reconstructed to generate a list of all intracellular metabolites synthesized or degraded in the metabolic network. The intersection of both lists (cyan bars) is passed to a third path (3) as candidates for docking simulations using the predicted protein structure. Predicted binding affinities that exceed some threshold are finally passed as candidates for experimental validation.

We propose a concept for a computational pipeline built on existing tools to progressively narrow the search space of potential transporter-substrate binding pairs. By limiting the number of candidate substrates for each predicted transporter structure, one can devise a strategy to limit compute resources and alleviate some of the scalability problem for downstream experimental validation. The pipeline (Figure 3), makes parallel use of bioinformatics, systems biology tools and molecular dynamics simulations to generate a short-list of substrates with relatively high predicted ligand binding affinities. The workflow begins with homology search against the TCDB to annotate genes to the lowest level of ontology, given some threshold alignment. Although the TC System is not phylogenetically structured *per se*, an analogous approach to “Lowest Common Ancestor” (e.g., MEGAN; Huson et al., 2007) could be used to assign ontology terms at a threshold confidence level. In this scheme, a gene with close sequence similarity to a transporter gene in the TCDB is annotated to level 5 (e.g., 2.A.1.1.1), whereas another with weaker alignment is annotated to level 3 (e.g., 2.A.1). Structuring the depth of annotation is a conservative strategy to generate a list of children substrates that the query structure could possibly transport (i.e., all substrates beneath 2.A.1). In a parallel step, a draft GEM is reconstructed, returning the full set of intracellular metabolites. By taking the intersection of these two lists, we pare down the candidate substrates to only those which the organism could conceivably take up or secrete. More stringent approaches exist at this step, including an analysis of uptake and secretion potential given the free exchange of all intracellular metabolites across the system boundary using flux variability analysis (Gudmundsson and Thiele, 2010), but the concept remains the same. Finally, from the intersection set, predictions of ligand binding affinity are used to generate a ranking of candidates. This step takes advantage of advances in structure prediction (e.g., AlphaFold; Jumper et al., 2021; RoseTTAFold; Baek et al., 2021), binding site inference, docking and molecular dynamics simulations (e.g., Ohnuki et al., 2023). One approach here is to infer transporter binding sites from homologus ligands and their cognate binding pockets already in the PDB databank (PDBspheres; Zemla et al., 2022). Fusion Docking-ML calculation can then be performed to determine the most favorable ligand poses in the transporter (Jones et al., 2021). If increased fidelity is desired, various versions of molecular dynamics simulations can be performed to qualitatively and/or quantitatively predict favorable dynamical protein-ligand interactions and associated binding constants (Sohraby and Nunes-Alves, 2023). This approach benefits from high throughput, with each simulation taking approximately 0.01 s/ligand (Zhang et al., 2014), but may suffer from the lack of sensitivity for low molecular weight ligands (less than 4 carbons) and metals, although progress is being made (c.f., zinc; Wang, 2023). An exciting development in this area is quantum docking simulations (Heifetz, 2020), which would, in principle, allow quantitation of binding affinities for these small molecules. The drawback with this quantum docking is throughput, with simulations taking on the order of minutes to hours depending on the size of the binding pocket, each. At this stage, depending on one’s objectives and the resources available, one might either submit the best candidates for experimental validation or simply apply a threshold affinity for annotation.

### 2.7 Prospects for transporter functional genomics

With the advent of reliable protein structure prediction tools such as AlphaFold (Jumper et al., 2021), we will likely see many of our current sequence-to-function annotation tools replaced by a whole new generation of sequence-to-structure-to-function tools over the next decade, both for enzyme annotation and for substrate-specific transporter annotation. However, the availability of large-scale substrate specificity data to train such tools will likely continue to be a bottleneck. While computational methods can pare down the search space of transporter-ligand binding candidates, evidence for transporter annotations should come from experimental validation, preferably *in vivo* (David et al., 2019). Recent advances in laboratory automation and mass spectrometry are dramatically increasing the throughput of functional and phenotypic screening (Coutant et al., 2019), and there is potential for functional genomics guided by mechanistic models. For instance, dynamic FBA can be used to identify target genes to generate smaller, metabolic process-specific deletion libraries for subsequent phenotyping (Brunnsåker et al., 2023). To our knowledge, these approaches have not yet been applied to transporters but could be easily adapted using Biolog-like screens (Bochner et al., 2001) or exometabolomics (Jenkins Sánchez et al., 2022). One high-throughput approach involves the use of a substrate-selective riboswitch as biosensors (Genee et al., 2016). When expressed along with metagenomic DNA fragments, transformants could be screened for their ability to grow on the substrate, and in so doing, the authors could assign function to uncharacterized transporters and identified numerous transporter annotations in error for multiple substrates. Another exciting recent development is Boundary Flux Analysis (reviewed in Lewis, 2024), a method to link changes in metabolite concentrations in growth media to constraints on uptake or secretion rates in GEMs. This approach appears scalable and holds great promise for screening deletion libraries.

## 3 Conclusion

Errors in transporter annotation arise from a variety of sources, most often resulting in missing or false assignments to substrates. Because of the non-unique mapping of genes to transporters to substrates, these errors metastasize, contributing to horrendous performance in the genotype-phenotype mapping of automated GEM reconstructions based on genome annotation alone. Mischaracterization of species-environment interactions is compounded when inferring microbial interactions in community models, leading to further expansion of spurious and false interaction predictions, and therefore poor fidelity to observations. To complement the progress enjoyed by other aspects of GEM reconstruction, we need to pursue new computational and experimental approaches to the transporter annotation problem. We offer a strawman workflow combining hierarchical ontology filtering with molecular dynamics simulations, and look to emerging high-throughput screening methods to validate predictions. Until the larger systems biology community and sponsors prioritize this challenge, we can continue to expect diminishing returns on advances in microbiome modeling.

## Data Availability

Publicly available datasets were analyzed in this study. This data can be found here: BIGG Models (http://bigg.ucsd.edu/) and Transport DB2 (http://www.membranetransport.org/).
